# Occurrence and potential mechanism of holin-mediated non-lytic protein translocation in bacteria

**DOI:** 10.15698/mic2022.10.785

**Published:** 2022-09-23

**Authors:** Thomas Brüser, Denise Mehner-Breitfeld

**Affiliations:** 1 Institute of Microbiology, Leibniz Universität Hannover, Hannover, Germany.

**Keywords:** holins, endolysins, toxins, Tat transport, bacteriocins, clostridia

## Abstract

Holins are generally believed to generate large membrane lesions that permit the passage of endolysins across the cytoplasmic membrane of prokaryotes, ultimately resulting in cell wall degradation and cell lysis. However, there are more and more examples known for non-lytic holin-dependent secretion of proteins by bacteria, indicating that holins somehow can transport proteins without causing large membrane lesions. Phage-derived holins can be used for a non-lytic endolysin translocation to permeabilize the cell wall for the passage of secreted proteins. In addition, clostridia, which do not possess the Tat pathway for transport of folded proteins, most likely employ non-lytic holin-mediated transport also for secretion of toxins and bacteriocins that are incompatible with the general Sec pathway. The mechanism for non-lytic holin-mediated transport is unknown, but the recent finding that the small holin TpeE mediates a non-lytic toxin secretion in *Clostridium perfringens* opened new perspectives. TpeE contains only one short transmembrane helix that is followed by an amphipathic helix, which is reminiscent of TatA, the membrane-permeabilizing component of the Tat translocon for folded proteins. Here we review the known cases of non-lytic holin-mediated transport and then focus on the structural and functional comparison of TatA and TpeE, resulting in a mechanistic model for holin-mediated transport. This model is strongly supported by a so far not recognized naturally occurring holin-endolysin fusion protein.

## NON-LYTIC HOLIN-MEDIATED TRANSPORT OF FOLDED PROTEINS–AN ALTERNATIVE TO THE Tat PATHWAY

There are two general protein translocation pathways in the cytoplasmic membrane of bacteria: (1) The general secretion (Sec) system for the transport unfolded proteins, and (2) the twin-arginine translocation (Tat) system for the transport of folded proteins [1]. Proteins that are transported by these systems are synthesized with N-terminal signal peptides that are important for the recognition by the transport systems and for the translocation mechanism. In diderm bacteria, proteins that are secreted into the environment need to cross the cytoplasmic and the outer membrane, and this transport can occur either in two steps, employing Sec or Tat systems for the cytoplasmic membrane and other pathways for the outer membrane, or it can occur in a single step by secretion systems that cross both membranes [2][3]. In monoderm bacteria, transport across the cytoplasmic membrane can already release a protein into the environment, if only the passage through the cell wall is enabled.

**Figure 1:**
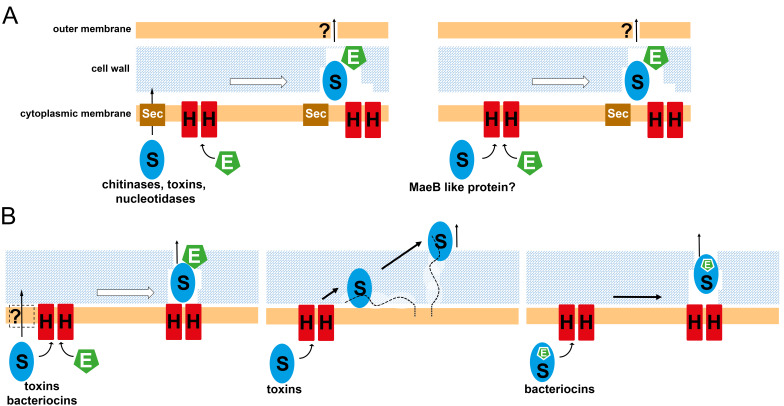
FIGURE 1: Schematic surveys of potential non-lytic secretion pathways that involve holin-mediated transport in Gram-negative (A) and Gram-positive (B) bacteria. Holins (H, red) are included with two protomers to symbolize that association of multiple protomers is expected to be required for transport. Examples for substrates (S, blue) of specific pathways are mentioned. Endolysins or endolysin domains are indicated (E, green), as is the Sec translocon (Sec, brown). Lipoteichoic acids are symbolized by a dashed black line. See text for more details.

Some extracytoplasmic proteins are neither transported by the general Sec or Tat pathways, nor by the recognized specific secretion pathways [4]. In several cases, such proteins are substrates of holin systems. Holins originate from phages where they serve to release endolysins to the cell wall, resulting in peptidoglycan degradation and cell lysis. There are seven large holin superfamilies known, as listed in the Transporter Classification Database [5][6], but in principle holins are divided in two classes: (1) canonical holins that transport endolysins directly, and (2) pinholins that can depolarize the cytoplasmic membrane, which results in a release of endolysins that are membrane-anchored by so-called signal anchor release (SAR) domains [7]. Accordingly, holins that are harnessed by bacteria to transport specific proteins are canonical holins. Canonical holins were named “holins” when they were proposed to form membrane lesions [8], and well-studied holins of Gram-negative bacteria have indeed been shown to generate the expected large lesions of the cytoplasmic membrane [9]. As such large lesions would not enable any specific transport but rather a non-specific release of cytoplasmic proteins, there have been doubts whether holins can be regarded as protein transport systems at all. This view changed within the last decade, as holins have been identified in Gram-negative and Gram-positive bacteria that are involved in a non-lytic transport of folded proteins that in principle could also be transported by the Tat pathway. Gram-negative bacteria are shown to use prophage-derived non-lytic holin/endolysin systems to locally permeabilize the cell wall for the secretion of proteins (termed type 10 secretion, [10]). In this review, all classes of mureinolytic enzymes that are transported by holins and therefore are closely related to phage holin/endolysin systems are termed “endolysin”. It is suggested but not known yet whether Gram-negative bacteria use holins also for the transport of other proteins. In principle, folded proteins could also be transported by the Tat system, and therefore the use of holin-mediated transport represents an evolutionary harnessing of an existing phage-derived system for cellular secretion purposes. Interestingly, at least clostridia-related Gram-positive bacteria that lack Tat systems apparently went one step further and employed holins also for the transport of toxins and bacteriocins (**Figure 1**). In some cases, an endolysin is likely to be a second substrate of the holin, which then can function to locally permeabilize the cell wall as in the case of Gram-negative systems. In other cases, the original holin-associated endolysin gene has been fragmented or deleted, and secreted proteins possibly use alternative pathways for the cell wall passage.

The size limit for efficient free diffusion of globular hydrophilic proteins through the cell wall of Gram-positive and Gram-negative bacteria has been calculated to be near 50 kDa [11]. For larger proteins, there are at least three possibilities to achieve this passage without cellular lysis (**Figure 1**): (1) endolysins can locally permeabilize the cell wall without destroying it, (2) the secreted protein itself contains a weak murein hydrolase activity, or (3) the secreted protein uses general cell wall remodeling pathways. While the first two options are self-evident, the third option needs some explanations. It is already known that changes in lipoteichoic acid structure can enhance the secretion of recombinantly produced proteins in Gram-positive bacteria [12]. It has been even shown that proteins can become trapped in the cell wall if there are no teichoic acids [13]. Teichoic acids thus likely cause irregularities in the murein network, interfering with the usual “tessera” mesh formation, and forming spots of lower murein density that facilitate the passage of proteins. In principle, teichoic acids are synthesized inside the cytoplasm and transported to the outer surface of the inner membrane [14]. While they are at their basis covalently attached to either lipids or murein (lipoteichoic acids vs. wall teichoic acids), their large polar tail consisting of diverse polyol phosphates or glycosyl polyol phosphates somehow traverses the peptidoglycan, most likely depending on murein remodeling processes that are influenced by teichoic acids. Proteins with teichoic acid-binding domains may therefore preferentially diffuse through these regions of low murein density, or they already interact with newly generated teichoic acids on the outer face of the cytoplasmic membrane and use this interaction for being shuttled to the surface of the murein by the cell-wall remodeling processes that permits the extension of the teichoic acids to the cell wall outer surface. As most of the teichoic acids become exposed to the surface, most proteins will eventually reach the surface. This is likely the pathway for the pneumococcal surface protein PspA that is usually anchored to cholin of lipoteichoic acids by its cholin-binding repeats [15]. PspA is transported by the Sec pathway and has a molecular mass of 67-98 kDa, depending on the strain [16]. As PspA is a surface-exposed protein that is released from the cell wall by cholin treatment or cholin deficiency [15], proteins with cholin-binding repeats may thus better penetrate the cell wall. Besides PspA, also streptococcal autolysins with lysozyme or amidase activity (which originate from prophage endolysins), and endolysins of pneumococcal or streptococcal phages, such as Cp-1 of oral streptococci, possess such cholin-binding repeats [17]. As we will see below, most large clostridial toxins that are likely transported by the non-lytic holin pathway do also contain multiple cholin-binding repeats and thus may use this pathway for the cell-wall passage (**Figure 1**). There may even exist a cholin-induced release of toxins from bacterial surfaces upon exposure to cholin-rich environments at intestine tissue. In the following two chapters we will briefly summarize the current knowledge about the use of holin-mediated transport for non-lytic secretion of proteins by Gram-negative and Gram-positive bacteria, before going into mechanistic details of the transport *per se*.

## NON-LYTIC HOLIN/ENDOLYSIN SYSTEMS FOR THE SECRETION OF CHITINASES AND TOXINS IN GRAM-NEGATIVE BACTERIA

*Serratia marcescens* secretes chitinases, and it has been nicely shown that this secretion depends on a non-lytic permeabilization of the bacterial cell wall by an endolysin, ChiX, which is secreted by the holin ChiW [18][10][19]. Accordingly, these proteins are genetically linked to the chitinases ([10], **Figure 1A**). While the chitinases cross the inner membrane via the Sec pathway, the endolysin is holin-dependently translocated, and its activity is most likely low and restricted to a specific point of the cell wall to avoid cell lysis. How the chitinases cross the outer membrane is currently unknown. Importantly, it could be shown by fluorescent protein fusions on individual cell level that the production of the holin and the endolysin occurred in a small subpopulation of the cells and did not result in lysis of these cells [19]. This is an important aspect that is currently better analyzed in case of the Gram-negative systems than in the Gram-positive systems. The transport deficiency in a strain lacking the holin could be complemented by Tat-dependent transport when the endolysin was fused to a Tat signal peptide, indicating that the holin served to transport the folded endolysin [19]. Genomic analyses indicated that Sec-dependently translocated chitinases are frequently associated with holin/endolysin systems that most likely similarly function to permit their passage through the peptidoglycan [10].

Another example of non-lytic transport of endolysins in Gram-negative bacteria comes from the secretion pathway of the typhoid toxin of *Salmonella enterica* Typhi [20][21]. This toxin requires the endolysin TtsA for the passage of the toxin through the cell wall, and TtsA and the toxin are genetically linked [20] (**Figure 1A**). Toxin secretion also depends on the unusual transpeptidase YcbB, which directly cross-links two diaminopimelic acid residues that are at position 3 of the murein peptides [22]. This so-called 3-3 or LD cross-link makes up only about 2 % of the murein cross-links and is found specifically at poles of cells. Extensive biochemical and structural analyses clearly demonstrated that TtsA has indeed a rare substrate-specificity to these LD cross-links [22]. Like in the case of the endolysin that is involved in the release of chitinases from *S. marcescens*, TtsA does not possess a signal peptide and must be holin-dependently translocated, representing another example for the harnessing of holin-endolysin systems for non-lytic transport. This view is supported by the finding that TtsA is genetically linked to holin genes at toxin-loci of other enterobacteriaceae [10].

Based on genomic co-occurrence and physiological arguments, also the cell-wall passage of Sec-dependently translocated nucleotidases has been suggested to depend on holin-mediated murein hydrolase transport [10]. Beside different types of endolysins that have distinct substrate specificities, also other proteins might be transported by holins in Gram-negative bacteria. In enterobacteriaceae, insecticidal toxins occur that miss signal peptides and that are clearly genetically linked to holin/endolysin pairs, and a holin-dependent transport of these has been considered [10]. The genetic linkage of the insecticidal tripartite toxin complexes Tc to holin/endolysin systems has been recognized in *Photorhabdus* and *Yersinia* species [23][24], and the release of this large ∼1 MDa toxin complex has been suggested to be mediated by cellular lysis as achieved by a lytic holin/endolysin activity [24]. A lytic release was further evidenced by the observation that also genetically linked spanins contribute to toxin release [23]. Spanins are described to be associated with outer membrane disruption for phage lysis [25]. This case may thus be an example for the use of a lytic holin/endolysin system for a toxin release, which then would require lysis of a subpopulation of the cells to be physiologically meaningful, but this issue remains to be experimentally clarified.

In addition, the holin responsible for the endolysin secretion that permits the cell wall passage of chitinases secreted by *Serratia* species has been found to be genetically also associated with a gene encoding a homologue of the NADH-dependent bifunctional malic/malolactic enzyme MaeB [26]. This protein contains no signal peptide but was found in the secretome of *S. marcescens*, and its secretion was reduced in a holin-deficient mutant strain, which is why it has been suggested to be substrate of the holin as well [19][10]. However, there is no evidence for a functional role of MaeB-like proteins outside the cytoplasm so far, and secretion of several other proteins that are not associated with holins has similarly been reported to be reduced in the holin deficient strain [19], suggesting that possibly not all of these proteins need to be transported by the non-lytic holin transport pathway.

## NON-LYTIC HOLIN-MEDIATED TRANSPORT IS INVOLVED IN THE RELEASE OF TOXINS IN CLOSTRIDIA

The first clear evidence for a non-lytic holin-mediated transport came from studies on the release of the large clostridial toxins (LCTs) TcdA and TcdB from *Clostridioides difficile*. Clostridia are Gram-positive, obligate anaerobic, spore-forming bacteria that often prefer sugars (saccharolytic clostridia) or amino acids (proteolytic clostridia) as substrates for growth, although they may also ferment both [27][28]. The gut of animals and humans is a perfect anoxic nutrient-rich habitat for clostridia. To get access to the nutrients coming from the intestinal wall and eventually from the complete host without being harmed by immune responses, some pathogenic clostridia developed strategies to kill host tissue and eventually the host. These clostridia produce a plethora of harmful toxins. Depending on the species and strain, untreated disease patterns range from diarrhea to death. Among the most important toxins are the LCTs, which are proteins of more than 200 kDa molecular mass that are so far known from *C. difficile, Paeniclostridium sordellii, Clostridium novyi*, and *Clostridium perfringens* [29]. Upon secretion, these toxins are taken up by host cells, where they exert their detrimental effects by glycosylation of signaling pathway proteins, but also by glycosylation-independent mechanisms, eventually leading to pyknotic cell death [30][31][32][33].

**Figure 2:**
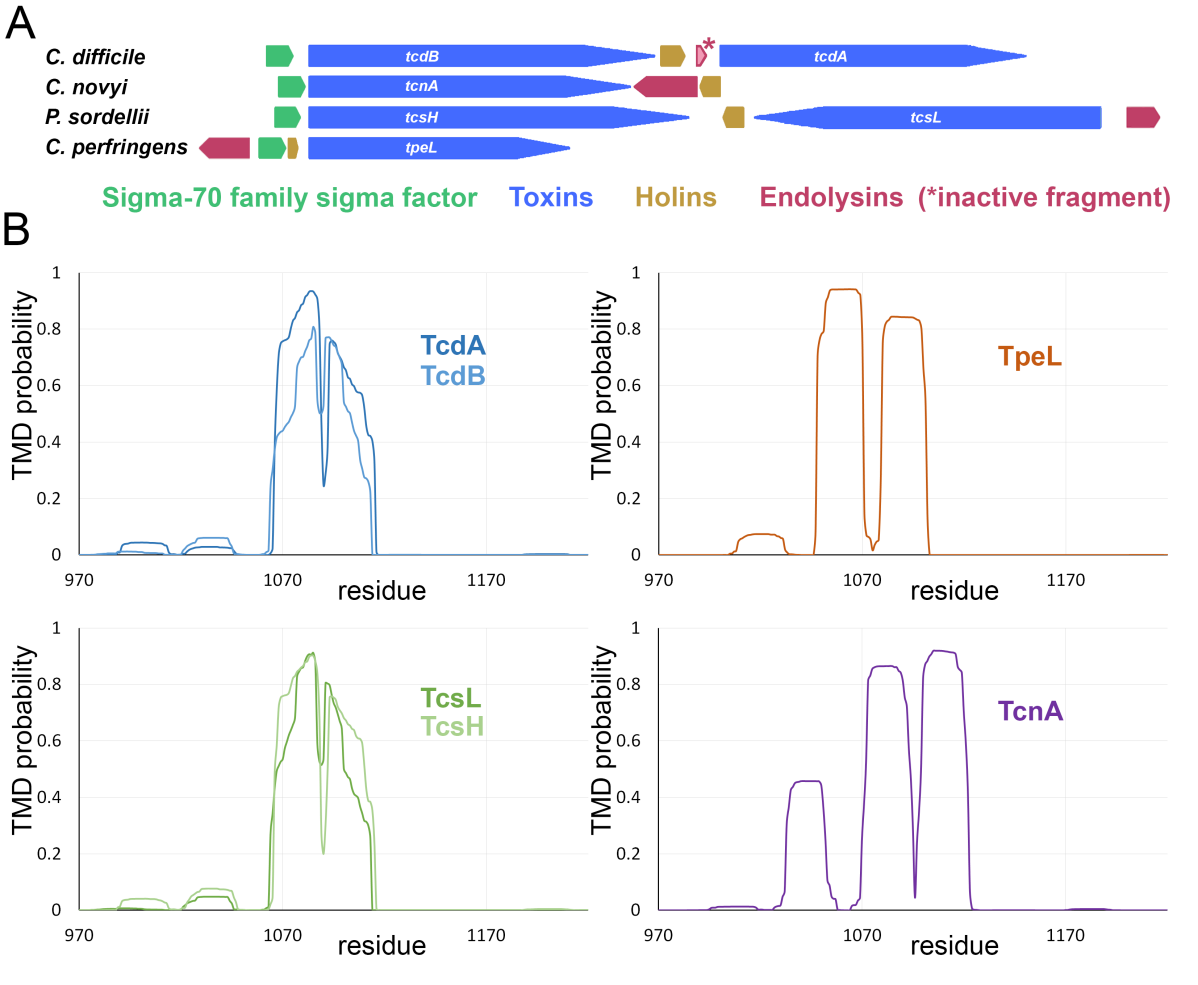
FIGURE 2: Large clostridial toxins (LCTs) are genetically associated with holin/endolysin systems and are unlikely to be compatible with Sec transport. (A) Genetic environment of the genes encoding the so far known LCTs (blue). Note the presence of genes encoding a sigma-70 family sigma factor (green) and a holin (ocher) in direct neighborhood of all toxin genes. Note also that endolysin genes, or in one case a fragment of an endolysin gene, are present at all toxin loci (red). (B) Detection of a hydrophobic region in the central part of all LCTs, which has sufficient length for a trans-membrane helix and therefore would be incompatible with Sec transport. Prediction of trans-membrane helices by TMHMM 2.0 [46], using the sequences of the indicated regions only. See text for more details.

Clostridia can release their toxins by two pathways: cellular lysis and non-lytic secretion. The **lytic pathway** has been found to account for most of the released toxins under conditions of growth limitation and/or spore formation [34]. In the specific medium that had been used in these experiments, lysis has been later shown to be induced by the Sec-dependently translocated autolysin, Cwp19, which is responsible for cell wall degradation during stationary phase in glucose-containing media [35]. In other media, unidentified autolysins may serve that purpose. First evidence for a **non-lytic toxin release** came from proteomic analyses [36], and Govind *et al.* recognized in 2012 that efficient toxin release during exponential growth depends on the holin TcdE [37] that is encoded at the pathogenicity locus (PaLoc) in direct vicinity to the genes for TcdA and TcdB (**Figure 2A**, [37][38]). No increased levels of other cytoplasmic proteins could be detected in culture supernatants, suggesting non-lytic transport. However, there remained the option that only a very small population of the cells produce very large amounts of toxins, which after lysis could appear like non-lytic release. To clarify this aspect, the regulator for PaLoc genes, TcdR, was constitutively produced, resulting in high toxin production already at early exponential growth. Under these conditions, toxins were clearly TcdE-dependently secreted without any indication of TcdE-dependent lysis in highly sensitive FACS analyses [39]. This experiment demonstrated non-lytic TcdE-dependent toxin secretion.

All holins that are genetically associated with the known large clostridial toxins are able to transport endolysins and are therefore bona fide holins [40][37][41][42]. In case of the holin TcdE, its phage origin could be traced back, and remnants of the associated endolysin gene have been found in direct vicinity of *tcdE* in the PaLoc of *C. difficile* [40]. The inactivation of the original endolysin gene at this locus supports the idea that a lytic endolysin transport may not be the purpose of the holin. Possibly TcdE might transport a prophage-derived endolysin that is encoded elsewhere in the genome, and this might support the cell wall passage of the toxin. However, this does not solve the problem of the membrane translocation for the toxins. In principle, toxin-associated holins could permit a non-lytic secretion of toxins. A holin-mediated transport of a toxin would require cytoplasmic folding of the toxin, and the toxins indeed fold inside the cytoplasm [43][44][45]. As the toxins lack signal peptides and as folding is not prevented, the toxins are not transported by the Sec pathway that is restricted to unfolded proteins with signal peptides. This may be due to an incompatibility of toxins with the Sec system: clostridial toxins usually contain a central hydrophobic region that could erroneously anchor these toxins in the membrane, thereby abolishing transport. The Hidden-Markov model based TMHMM algorithm [46] attributes only a very small probability (<20%) for a trans-membrane segment when the full-length toxins are analyzed, thereby correctly assigning the toxins as soluble proteins. However, when the analysis is restricted to the region of the hydrophobic segments and their surroundings, these hydrophobic segments are clearly assigned trans-membrane regions (**Figure 2B**). The different predictions are due to the very large extracytoplasmic domains of the full-length protein and the global view of a soluble protein that is generated by TMHMM, which outcompetes the trans-membrane probability (Anders Krogh, personal communication). If the toxins were transported by the Sec pathway in an unfolded state, such hydrophobic regions would block secretion. As there does not exist a Tat pathway for the transport of folded proteins in clostridia, there remains no known pathway for toxin transport other than the holin pathway. The strict genetic link to holins, the inactivation of the original endolysin gene, the absence of a signal peptide, the cytoplasmic folding, and the report of secretion under non-lytic growth conditions altogether argue for holin-dependent transport of toxins [37][40][39]. Furthermore, there is some evidence that a surface-fragment of the original endolysin can be produced that interacts with the toxin TcdB, which might mediate the interaction of the toxin with the holin [40]. Finally, it has been shown that cells with deleted toxin genes tend to lyse in the absence of the toxins, which nicely indicates a direct interaction of the toxins with the holin that either prevents large hole formations or competitively inhibits transport of endolysins that are encoded in prophages [37]. As a prophage-derived endolysin could also be transported by the system, it is possible that small amounts of transported endolysins locally permeabilize the cell wall to permit the passage of toxins. A second contribution to the cell wall passage may come from the “combined repetitive oligopeptide” (CROP) domain at the C-terminus of all known toxins except TpeL from *C. perfringens*. The CROP domain can contact host cell receptors at cell surfaces and is therefore currently believed to be involved in toxin uptake. However, other parts of the toxins also interact with receptors. The repeats in the CROP domain are “choline-binding modules” and thus may facilitate a teichoic-acid dependent cell wall passage as described above (chapter 1).

In *C. perfringens*, transport of the large toxin TpeL depends on the holin TpeE [42]. Like in the case of the other large clostridial toxins, the toxin has no signal peptide and therefore is not transported by the standard Sec pathway. The holin specifically is required for the toxin transport, and as recombinant TpeE alone sufficed for TpeL secretion, it has been suggested that the holin may transport the toxin directly [42]. Also the holin of *P. sordellii* has been found to specifically promote toxin release in that organism [41]. It has not been shown directly that the toxins themselves are transported by the respective holins, which is why the authors carefully claimed only a function in the release of the toxin, but, like in the case of *C. difficile*, the toxins lack signal peptides for Sec transport, are genetically linked to holins on pathogenicity loci, and there are no known alternative pathways for the non-lytic release of folded proteins in these clostridia. It is thus very likely that all large clostridial toxins can be transported by their respective genetically linked holin, and as these holins can also transport endolysins, such an endolysin transport may permeabilize the cell wall for the toxin release – similar to the known examples in Gram-negative bacteria.

## ALSO BACTERIOCINS MAY BE HOLIN-DEPENDENTLY RELEASED IN SEVERAL CLOSTRIDIA

**Figure 3:**
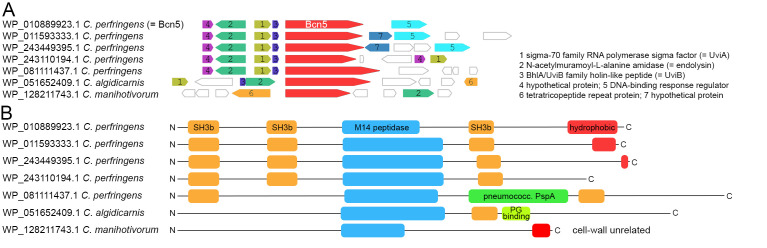
FIGURE 3: Secreted bacteriocins of the Bcn5 family are associated with holin and endolysin genes and can be recognized by the presence of SH3b domains. (A) Environment of genes encoding Bcn5 and six homologs. Note that the homologs of *C. perfringens* and *C. algidicarnis* strains are likely to be secreted bacteriocins. (B) Domain analysis of these Bcn5 homologs. SH3b domains as well as a PspA domain and peptidoglycan-binding domains (PG domain) are indicative for a function in the cell wall and hence transport. Note that the Bcn5 homolog of *C. manihotivorum*, which lacks an association with an holin gene, has none of these domains that would be indicative for a function outside the cytoplasm. See text for further details.

Another likely substrate for holins in clostridia are bacteriocins of the Bcn5 family, the prototype of which is Bcn5 that is encoded on the pIP404 plasmid of *C. perfringens* [47, 48]. Bacteriocins of this type are encoded on chromosomes and plasmids of many strains of *C. perfringens* and *Clostridium algidicarnis*, where they are always associated with a sigma factor (UviA), a BhlA-family holin (UviB), and an N-acetylmuramoyl-L-alanine amidase (endolysin) (**Figure 3A**, [49]). In *C. perfringens*, an endolysin is encoded upstream of *uviA* in the opposite direction, whereas in *C. algidicarnis*, the endolysin gene can be found also in an operon with the holin gene, directly followed by the gene of the bacteriocin, further supporting the functional relation (**Figure 3A**). Only the prototype Bcn5, encoded on plasmid pIP404, has been studied to some extent and there is little known about its homologs. Bcn5 and its homologs have no Sec signal peptide and thus face the same transport problem as do the clostridial toxins. The strict association of the bacteriocin with holins and endolysins suggests that secretion of Bcn5-family bacteriocins depends on the holin and the endolysin. The holin UviB either transports only the endolysin and thereby promotes cell lysis to release the bacteriocin, or it transports both the endolysin and the bacteriocin, and in this case secretion can take place without any lysis, as has been found for the toxins. Like large clostridial toxins, bacteriocins of the Bcn5-type are likely incompatible with Sec transport. Bcn5 and several of its homologs possess a very hydrophobic domain at the C-terminus that precludes a Sec-transport, but cytoplasmic folding might be required also for other reasons (chaperones). In case of the bacteriocins it makes much sense that bacteria do not lyse for the sake of killing other bacteria, and although more research has to be done on this aspect, it can be expected that Bcn5 is non-lytically secreted.

Bcn5 has a molecular weight of 96 kDa and is proposed to act on target cells by means of its C-terminal hydrophobic domain, which has characteristics of colicins that permeabilize bacterial membranes [47]. However, we found that the hydrophobic C-terminal region is highly variable among Bcn5 homologs, even missing in some cases, suggesting that other domains may also contribute to bactericidal effects. All Bcn5 homologs are predicted to possess a Zn-metallocarboxypeptidase domain that could exert such a function (**Figure 3B**). Specifically, the region from position 329 to 485 (Bcn5 numbering) is a M14 peptidase-like domain with similarity to the *Escherichia coli* murein peptide amidase A (MpaA, score of 71.5, e-value of 1e-13 in NCBI conserved domains database). MpaA catabolically hydrolyzes the γ-D-glutamyl-meso-diaminopimelic acid (γ-D-Glu-Dap) bond in murein tripeptides inside the cytoplasm [50], and it is intriguing that the Bcn5-family bacteriocins therefore likely have a mureinolytic activity. As *Clostridium* and *Bacillus* species cross-link their peptidoglycan using diaminopimelic acid, similar to *E. coli*, and do not use lysine as do other Gram-positive bacteria [51], a γ-D-Glu-Dap-hydrolyzing activity in the secreted bacteriocin might serve to facilitate the passage through the bacterial cell wall. However, as mentioned above, it is more likely that the cell wall passage is mediated by the endolysin, just as in the cases of holin-mediated secretion in Gram-negative bacteria. Alternatively, the hydrolytic activity of Bcn5 could be required by the bacteriocin to reach the target cell cytoplasmic membrane, which then can be perforated by the hydrophobic C-terminus. Interestingly, as mentioned above, not all Bcn5 homologs possess a hydrophobic C-terminal domain (**Figure 3B**) and therefore the amidase activity may also represent a bactericidal activity on its own. We also noted that bacterial SH3 (SH3b) domains are present in Bcn5 and its homologs when the bacteriocin gene is directly associated with a holin/endolysin couple (**Figure 3B**). SH3b domains are indicative for a function in concert with murein hydrolases, as shown for the cell wall hydrolytic NlpC/P60 family proteins [52], and therefore the bacteriocin homologs that do contain SH3b domains in addition to the peptidoglycan hydrolytic domain need to cross the cytoplasmic membrane to reach a cell wall. We found that more remote Bcn5 homologs exist that lack SH3b domains or any other hint to peptidoglycan interaction, and that are not associated with holins (e.g. in case of *Clostridium manihotivorum*), and therefore are likely cytoplasmic catabolic enzymes, similar to MpaA from *E. coli* (**Figure 3AB**).

In summary, it can be concluded that holin-dependent non-lytic protein transport exists in Gram-positive as well as in Gram-negative bacteria. In Gram-negative bacteria, non-lytic holin transport of endolysins enables the passage of secreted proteins such as chitinases and toxins through the cell wall. So far, no other proteins than endolysins have been shown to be holin-dependently translocated in Gram-negative bacteria. Non-lytic holin-mediated transport of endolysins occurs also in Gram-positive bacteria, which apparently use holins also for the non-lytic secretion of large clostridial toxins during exponential growth. A holin-dependent translocation is likely also at work in case of some clostridial bacteriocins. Holin-mediated transport may be the only possible pathway for the non-lytic secretion of folded proteins in clostridia, as these bacteria do not possess Tat systems.

## LEARNING FROM TatA, A Tat TRANSLOCON COMPONENT THAT RESEMBLES THE HOLIN TpeE

**Figure 4:**
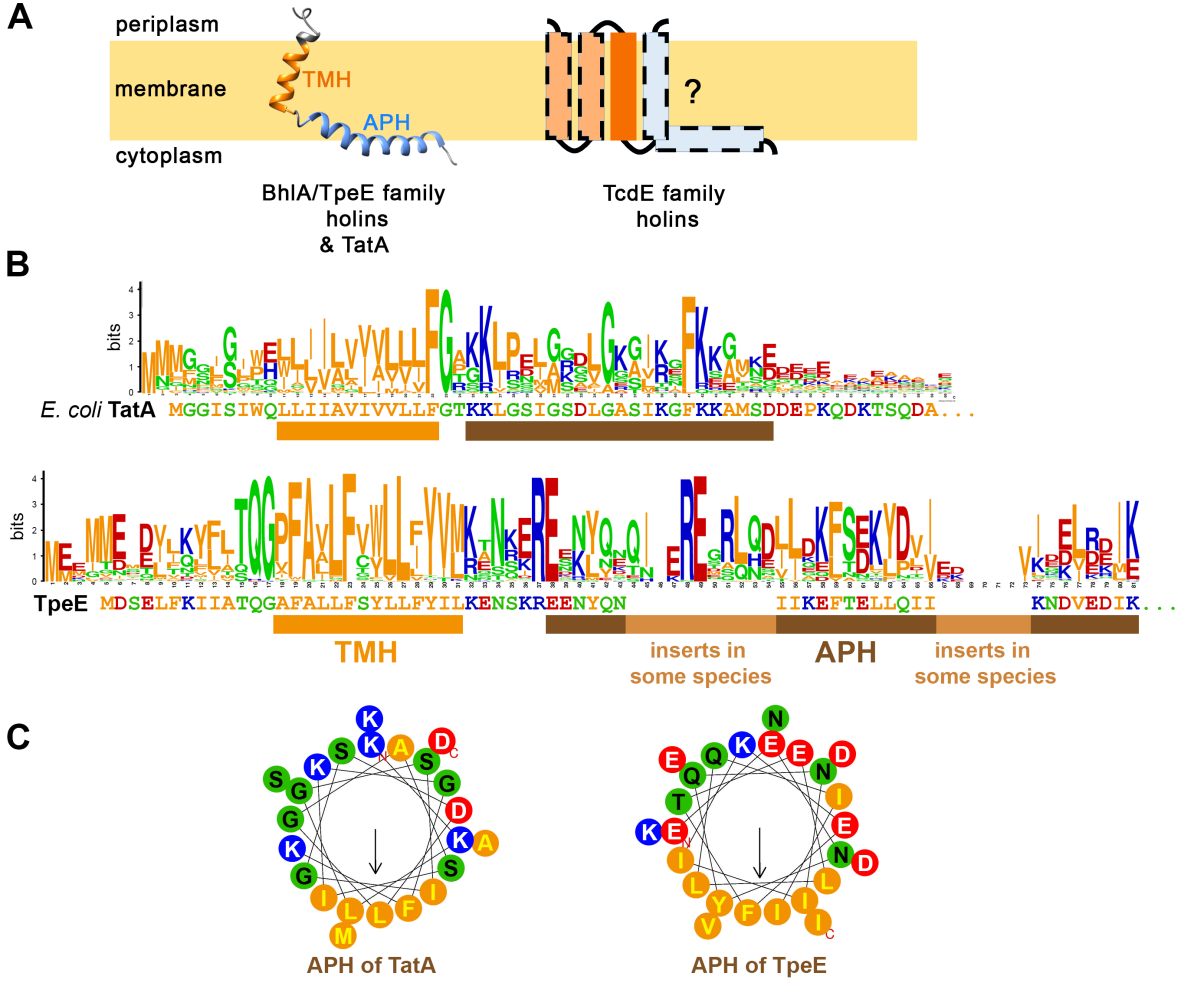
FIGURE 4: Structural comparisons of holins and TatA. (A) Schematic topology or BhlA/TpeE family holins and TatA in comparison to predicted topologies of TcdE. Note that TcdE homologs have always one TMH in common but can have up to three more predicted TMHs, resulting in a largely unclear topology. (B) WebLogo analysis of TatA and TpeE sequences [101], indicating conserved regions and the general characteristics of the residues (hydrophobic, orange; hydrophilic but uncharged, green; positively charged, blue; negatively charged, red). The corresponding sequences of *E. coli* TatA and *C. perfringens* TpeE are shown underneath the WebLogo diagram. The short consecutive hydrophobic region of the TMH is underlined in orange, the amphipathic helix (APH) is underlined in brown. The sequence alignment for the WebLogo-analysis has been made by Clustal-Omega [102] with 40 TatA sequences from very diverse organisms (α- β -, γ - δ -, and ε -Proteobacteria, Firmicutes, Actinobacteria, Cyanobacteria, Chloroflexaceae, Thermodesulfobacteria, and Aquificae), and with 100 TpeE homologs (TpeE homologs are only found in Firmicutes). (C) Comparison of the APHs of *E. coli* TatA and *C. perfringens* TpeE by helical wheel views. TMH predictions were done by TMHMM2 [46], and helical wheel views and APH visualizations were done using HELIQUEST [103].

While the toxin-transport related holins in *C. difficile, P. sordellii*, and *C. novyi*, as well as the known chitinases- or toxin-transport related holins of Gram-negative species are polytopic membrane proteins with variable predicted topologies, the holin TpeE from *C. perfringens* is very small (ca. 8 kDa) and contains only one transmembrane-helix (**Figure 4A**, [42]). TpeE belongs to the BhlA (bacteriocin-related holin-like) family of holin-like proteins (TCDB 1.E.27, [6]) that often occur in Firmicutes [42], whereas the other holins belong to the TcdE-family of holins (TCDB 1.E.19), with quite diverse members in many distinct phyla. Holin function has been previously shown for BhlA from *Bacillus licheniformis* and *B. pumilus* [53][54]. The predicted topology of TpeE resembles that of TatA, the membrane-permeabilizing component of the twin-arginine translocation (Tat) pathway for the transport of folded proteins in prokaryotes and eukaryotic organelles of bacterial origin [1]. Both are similar in size and possess a single N-terminal transmembrane helix (TMH), followed by an amphipathic helix (APH) (**Figure 4A**). As a charge zipper model has been proposed for the function of TatA in the past [55], such a mechanism has been suggested to be functional also in the case of TpeE [42]. However, more recent data argue against the charge-zipper mechanism for TatA [56]. Much more work has been done on TatA, and mechanistically important aspects can be revealed when the holin TpeE and the Tat component TatA are compared in more detail.

It therefore is important to briefly recapitulate our current knowledge. Besides TatA, minimal Tat systems require only one further component, which is TatC [57][58]. TatC recognizes the eponymous twin-arginine motif in signal peptides at the N-terminus of the transported proteins (the Tat substrates). Multiple copies of TatA associate with substrate-engaged TatC components, interact with the mature domain of the substrate (which is the usually folded region that follows the N-terminal signal peptide), and generate the environment for the membrane passage of the Tat substrates [59][60][61][62].

Most Tat systems require a third component, TatB, which is a second TatA-family protein that evolved to bind TatC more tightly [1]. In fact, most Tat studies have been done with such three-component Tat systems of *E. coli* and plant plastids. Structures of detergent-solubilized TatA and TatB monomers and of two interacting TatA protomers have been solved by NMR [63][64][65][66]. Also, TatC structures have been determined, in this case by crystallography [67][68]. While TatC is a polytopic membrane protein with six TMHs, TatA and TatB have a single TMH close to the N-terminus that is followed by an APH. TatB can have further helical structures in its longer C-terminal domain – at least this has been seen in case of the detergent-solubilized monomer whose structure has been solved [63] – but these parts of the protein are not essential for functionality [69]. We will now focus on TatA.

The N-terminal TMH of TatA is positioned very close to the end, with only 8-10 residues on the N-terminal side of the transmembrane helix (**Figure 4B**). These N-terminal residues are on the periplasmic side of the cytoplasmic membrane [70]. Longer N-terminal extensions rarely occur, and in one case it has been shown that a processing of such an extension was required to activate TatA [71]. The 8-10 N-terminal residues of active TatA are a mixture of small and flexible residues (Gly, Ser), other polar residues, and several aliphatic residues (**Figure 4B**). These residues are somehow related to the functional differentiation of TatA and TatB, as single mutations in this region can compensate for the absence of TatB [72]. Only the last residue of this region, the polar residue that precedes the hydrophobic transmembrane helix, is essential for the general activity [73]. In plants, this residue is always negatively charged and important for the reversible interaction with TatBC complexes [74]. Also, in two-component Tat systems this residue is usually negatively charged. In three-component Tat systems, this residue is polar but not necessarily charged [75]. It is important for hydrogen-bonding interactions with TatC, which are the basis for the assembly of multiple TatA protomers to TatBC core complexes [76].

The following TMH of TatA is very unusual: Being too short to span a membrane of normal thickness, it generates a hydrophobic mismatch with the membrane [75][77]. It consists of only 12 hydrophobic residues, flanked by the above-mentioned polar residue at the N-terminal side and a strictly conserved glycine residue on its C-terminal side (**Figure 4B**). While the last residue of the hydrophobic transmembrane helix is a conserved phenylalanine, the other residues are not conserved and are usually aliphatic. None of the hydrophobic residues is essential as they all can be exchanged by cysteine without losing functionality [73]. The hydrophobic mismatch of this TMH has been shown to generate membrane stress by thinning the membrane in case of multiple clustered TatA protomers [78][75][77]. MD simulations indicated that the adjacent APH plays an important role for this membrane thinning, as laterally aligned APHs remove lipid head groups from the region, which disturbs the lipid bilayer and causes the formation of a deep V-shaped groove in the membrane [75]. This is most likely the basis for the membrane destabilization that is believed to permit the membrane passage [79][80]. It is known that multiple TatA protomers cluster at active Tat systems and there is considerable evidence for an interaction of transported proteins with TatA during this membrane passage, which agrees with that model [59][60][61][62]. The membrane passage is associated with significant proton flux through the membrane [81], which likely is not triggering transport and therefore has been suggested to represent proton leakage that cannot be avoided when folded proteins cross the membrane [82][83]. This view is based on the apparent absence of a defined route for protons through the translocon that could energize protein transport [1], and on the observation that it seems to be the electric potential rather than the proton gradient that is required for transport, in the *E. coli* system [84].

Adjacent to the TMH, the two next residues form a hinge that connects the TMH with the APH. In this hinge, the glycine residue is strictly conserved and essential (**Figure 4B**, [73]), indicating that an unusual angle or flexibility between the secondary structures at this position is important for functionality. The following APH is highly important, and, in contrast to the TMH, many specific residues in the APH are essential for functionality [73]. Among these essential residues are highly conserved positions, such as a positive charge at the beginning of the APH, a glycine close to the center of the APH, and an “FK”- motif close to the end of the APH (**Figure 2A**, [82]), but also a considerable number of less conserved positions. Nobody knows yet what these essential positions are exactly required for, but MD simulations suggest that they may be involved in lateral associations of the APH [75], and they may also be involved in interactions with the mature domain of substrates [78][85]. The APH has a tilt orientation relative to the membrane [86], and MD simulations indicate that the APH significantly contributes to the trans-membrane anchoring of TatA [75].

On the C-terminal end of the APH, TatA possesses a region with several negatively charged residues. The removal of three of these negative charges did reduce but not completely abolish Tat activity, unless they were exchanged by hydrophobic residues [87]. The helical wheel projection of the TatA APH shows the strongly aliphatic characteristics of that helix (**Figure 4C**). The remaining C-terminal region of TatA is unstructured and not required for activity [69].

## TpeE IS UNRELATED TO TatA BUT MAY USE A SIMILAR MECHANISM FOR MEMBRANE PERMEABILIZATION

A direct comparison of TpeE with TatA immediately reveals that – despite their similar organization of secondary structural elements – they strongly differ in sequence, including those positions that are known to be essential for TatA activity. The N-terminal region of TpeE that precedes the TMH is with 13-14 amino acids a little longer than the corresponding region of TatA and similarly consists of a mixture of hydrophobic and polar residues. However, while charged residues are very rare at the N-terminus of TatA, they are conserved in the corresponding region of TpeE. TpeE homologs have up to three charges in that region, which usually has a negative net-charge (few are neutral). A negative net-charge could support the flipping of the N-terminus to the extracytoplasmic face of the membrane during membrane insertion. The C-terminal end of the TMH is usually flanked by a positively charged residue (Lys/Arg), in agreement with the positive inside rule [88], which would help in positioning the helix in the right orientation. In case of TatA, the immediately following APH with its positive charges at the N-terminal end may serve the same purpose.

TpeE has a highly conserved T-Q-G motif that is absent in TatA (**Figure 4B**). This polar motif marks the end of the N-terminal region, being followed by the hydrophobic TMH. The TMH of TpeE has a very short length of only 14 residues, similar to TatA with its 12 residues. It therefore immediately comes into mind that an APH might compensate for that short length – as it does in case of TatA – and indeed the TMH of TpeE is followed by an APH that could fulfil that purpose. But before discussing the APH of TpeE, it is worth to have a closer look on the specific amino acid sequence of the TMH of TpeE.

While TatA has little sequence conservation in its TMH, except a strictly conserved Phe at its very C-terminal end, TpeE shows considerably higher sequence conservation in its TMH. The TMH consensus sequence as derived from 100 TpeE homologs with sequence identity of 50-100% is P-F-A-X-L-F-X-W/Y-L-L-F/I-Y-V-M/L (**Figure 4B**). This is a high degree of conservation in comparison to the TatA TMH, which contains only one conserved residue (the Phe at its end). However, one has to keep in mind that all these TpeE sequences originate from Firmicutes, whereas the TatA sequence logo integrates much more diverse organisms from many phyla. Nevertheless, the degree of conservation in the TMH of TpeE clearly is higher than in other parts of the protein, which contrasts the situation in TatA. An unusual characteristic of the TMH are conserved aromatic positions. Normally, stable TMHs are rich in residues with aliphatic side chains, and the aromatic residues Tyr or Trp are preferentially found at an end of the helix, whereas Phe is found preferentially in the central part [89][90]. However, TpeE homologs possess four conserved aromatic positions that are scattered over the TMH, including a central W/Y position, indicating an unusual functional role that distinguishes this TMH from the TMH in TatA.

TpeE homologs contain multiple charges on both sides of the TMH, and there is no positive net-charge at the N-terminal end of this TMH, which is why the N-terminus - as in the case of TatA - likely is on the outer side of the cytoplasmic membrane. TatA inserts spontaneously into the membrane, even when added as purified protein [91], but TatA homologs usually have no or only a single negative net charge at their N-terminus [82]. It is thus likely that also TpeE can spontaneously insert into membranes.

In the TMH of TpeE homologs, the aromatic residues could promote strong helix-helix interactions and the hydrophobic mismatch that is generated by the clustered short helices would cause membrane thinning and destabilization. In fact, the TpeE homolog BhlA and its TMH alone have been found to have bactericidal effects when recombinantly overproduced, supporting the idea that the TMH can destabilize membranes [54]. This resembles the membrane-destabilizing effect of the short TMH of TatA [78]. As Gram-negative bacteria possess a protective outer membrane that prevents access of the peptide to the cytoplasmic membrane, it is logic and self-evident that the bactericidal effects were restricted to Gram-positive bacteria. In this context, it is interesting that the small holins have been proposed to resemble toxins of bacterial type I toxin-antitoxin systems, which are believed to destabilize cytoplasmic membranes [92][93].

Another remarkable characteristic of all TpeE-homologs is a K/R-X-N-X-E-R-E motif that immediately follows the TMH. There is never a hydrophobic residue in this sequence. We cannot predict whether or not this region can form a flexible hinge, but a conserved Gly such as found in the TatA hinge is missing in TpeE and its homologs. This may relate to the fact that a reversible orientational change of the APH relative to the TMH is not required in case of TpeE (see below). The conserved residues in this motif, as well as the following APH, may in principle be able to form hydrogen bonds and ionic interactions with neighboring TpeE protomers and/or transported proteins, supporting clustering, membrane destabilization, and transport.

The APH of TpeE homologs starts near the strictly conserved “RE”-Motif and contains many conserved positions that all differ from those conserved in the TatA APH. As the APH must be exposed at the surface of the membrane, it may play a role in substrate interactions, like in the case of TatA [85][78]. While the dimensions and properties of the APH from TpeE and TatA are similar, there is no similarity on sequence level, and TpeE homologs show a high degree of sequence variability in their APH, and some homologs even contain insertions (**Figure 4C**). This variability may well reflect an adaptation to transported substrates.

Together, the very similar structural organization – a short TMH followed by an APH – suggests a highly similar membrane-weakening mechanism as basis for TpeE- and TatA-mediated protein transport. Assemblies of multiple TpeE protomers likely destabilize membranes very similar to TatA assemblies that have recently been analyzed [75]. However, beyond this common principle, the more detailed sequence analysis points to major differences, which likely relate to the translocation steps that follow the substrate interactions with the membrane-destabilizing assemblies of TpeE or TatA. The following chapter will address these differences.

## TpeE-FAMILY HOLINS NEED TO CATALYZE TRANSPORT ONLY ONCE, WHEREAS TAT SYSTEMS REQUIRE A STRUCTURAL RESET THAT PERMITS REPEATED TRANSPORT

The main functional difference between holins and Tat systems is the fact that Tat systems need to function continuously during growth, whereas the holins evolved to transport endolysins that catalytically hydrolyze the cell wall and ultimately kill the cells. In case of holins, a disruption of the cytoplasmic membrane therefore does not need to be avoided or may even be a desired side effect. Mechanistically, both systems need to permeabilize membranes at the point of substrate transport, but while holins can in principle undergo one major, irreversible domain movement for the transport of a single substrate, the domain movements of Tat systems need to be tightly controlled and balanced to permit conformational reversibility in the membrane.

**Figure 5:**
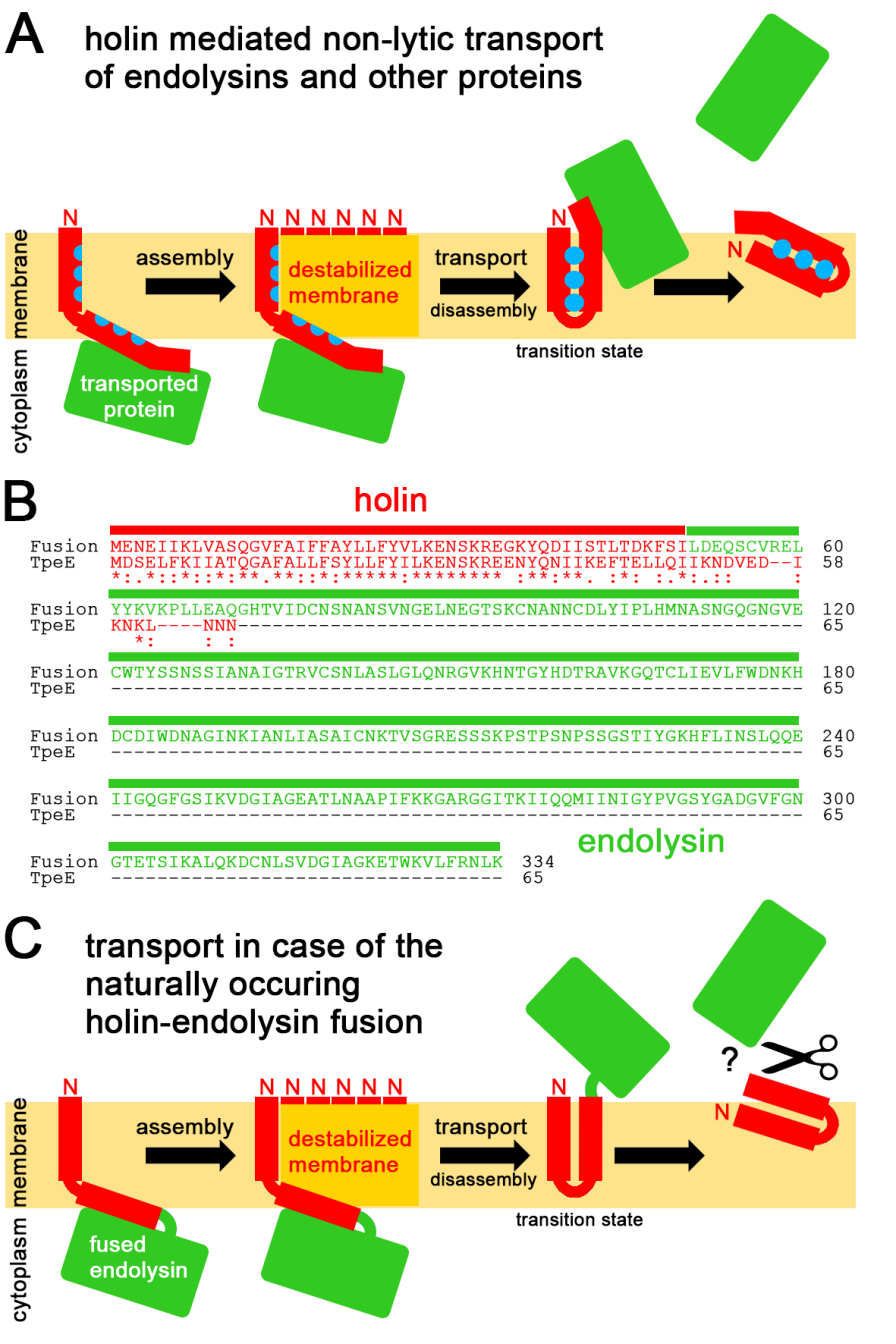
FIGURE 5: Mechanistic model for holin-mediated non-lytic transport of proteins. (A) We propose the following mechanism: Transported proteins associate with the cytoplasmically exposed APH/C-terminus region, holins assemble to multimeric patches that locally destabilize the membrane by membrane-thinning as in case of TatA (assembly; note that for clarity reasons only the multiple N-termini of the associated protomers are indicated), the hydrophobic face of individual substrate-associated APHs in these multimeric holin patches flip at these destabilized membrane sites irreversibly into a trans-membrane orientation, triggered by interactions with the TMH, thereby moving the associated protein through the destabilized membrane (transport), the interacting TMH and APH are now not anymore stably trans-membrane oriented and dissociate from the other holin protomers (disassembly), resulting in an unstable transition state as the hydrophilic face of the APH will readily move to the membrane surface, finally resulting in release of the transported protein from the trans-side of the membrane. (B) Sequence and domain organization of the natural holin-endolysin fusion, and alignment with *C. perfringens* TpeE. Holin sequences are in red, the fused endolysin sequence is in green. (C) Translocation mechanism as expected for the fusion protein. The mechanism as described in (A) is strongly supported by the natural holin-endolysin fusion. The fused endolysin might need to be cleaved off to function. See text for details.

This difference between holin- and Tat-mediated transport may well be the reason for the above-described major structural differences between the TpeE-family holins and TatA. For TatA, it is clear that the L-shaped topology is retained, and the C-terminus remains on the cytoplasmic side [94]. It once has been proposed that the APH of TatA might adopt a transmembrane topology during transport [55], but the energetic barrier to flip back would be very high, and later *in vivo* experiments did not support the model [56]. In case of TpeE-like holins, the predicted L-shaped topology is similar, but there is no special conserved flexible residue in the hinge region, and this may be because these holins do not require the APH to move reversibly, i.e. the APH does not need to remain on the cytoplasmic face of the membrane. The high conservation of several positive and negative charges in the APH suggests that salt bridges are important for the assembly and/or mechanism of the functional holin. As phage holin systems can afford being inactivated by one translocation event, it is indeed possible that the APH irreversibly flips, thereby channeling a C-terminally bound substrate through the membrane bilayer (**Figure 5A**). This flipping would be facilitated by the thinned and disordered membrane in the presence of multiple associated holins, and interactions of the hydrophobic side chains of the APH with the TMH might play a crucial role (symbolized by blue dots in **Figure 5A**), which would explain the high degree of conservation of specific hydrophobic residues within the TMH and the APH. Like the case of TatA [75], associations of multiple, possibly >20 holin protomers are likely required to achieve efficient membrane destabilization of dimensions that suffice to translocate larger proteins. Moreover, it is likely that the association of multiple holins to translocation-compatible patches is triggered by (or may even require) interactions with the substrate, which could avoid prolonged proton leakage. After the flipping event, the holin likely dissociates from the assembly, as the contacts of the APH with laterally aligned other APHs of neighboring protomers are lost, and due to the change in topology the protein can perfectly accommodate in membrane regions of normal thickness. The TMH interacts with the hydrophobic face of the APH, but the hydrophilic face of the APH will be highly unstable in the membrane environment and most likely immediately moves to the outer surface of the membrane (**Figure 5A**). Such a movement would prevent continued proton leakage, which otherwise could damage the cells. Alternatively, membrane protein quality control proteases might remove holins after transport, as they remove denatured proteins from the membrane. The membrane-bound quality control AAA+ family metalloprotease FtsH is known to serve such purposes in bacteria, mitochondria and plastids [95][96]. As this protease is conserved in clostridia, it may be responsible for a removal of holins after transport.

Such a “membrane-weakening and flipping” mechanism would function best if the folded cargo protein is somewhere bound to the C-terminal end of the APH. In such a constellation, the cargo protein would be dragged through the membrane by the flipping event, and no other component would need to be involved, which is a very important difference between the holin and TatA. TatA must avoid an irreversible flipping and thus requires TatC to pull the substrate through the weakened membrane. TatC inserts the signal peptide into the membrane and thereby exerts a pulling force that aids in the translocation process without making irreversible movements necessary [97]. In contrast, TpeE can afford an irreversible flipping and thus does not depend on further protein factors for transport. Other holins of the cluster could in principle move in a concerted way, but for steric reasons it appears more likely that other holins are simply pushed aside during transport and move back thereafter, and that the flipping occurs only at one or few holin protomers within larger holin assemblies. Therefore, there is no major membrane disruption required for this kind of transport. Lipids and lipid disorder likely play a role, both for enabling transport as well as for sealing the translocation site after passage of the cargo protein.

What alternatives exist for the herein proposed “membrane-weakening and flipping” mechanism? Non-lytic holin-mediated transport could in principle be achieved also by gated pore mechanisms or by other types of substrate-selective hydrophilic pores that are formed by defined arrangements of holins only. Such usual mechanisms have also been postulated for TatA in early years [98, 99], before the “membrane-weakening and pulling” hypothesis was published [79], and before TatA truncational analyses [69], the demonstration of transport with signal peptides cross-linked to TatC [100], NMR structures [66], the demonstration of membrane-weakening [78][77], and MD simulations [75] argued against these older models. To achieve an autonomous transport of folded proteins, usual transporters would require a large size with domains for a large channel, gating, energy coupling etc., and as even the largest holins are rather small membrane proteins, it seems unlikely that holins are large enough to enable such mechanisms. For the small holins such as TpeE, such mechanisms are unrealistic. As the same endolysins have been shown to be translocated by holins of distinct families (e.g. [41]), and as similar holins likely can transport highly distinct protein substrates (such as toxins or bacteriocins), holins most likely use a conserved, yet to be identified general recognition principle. The “membrane-weakening and flipping” mechanism directly links recognition to translocation, and therefore similar mechanisms may be implemented by distinct holins to carry out non-lytic translocation, possibly involving a flipping of C-terminal domains.

## THE C-TERMINAL FLIPPING MECHANISM IS SUPPORTED BY NATURALLY OCCURRING HOLIN-ENDOLYSIN FUSIONS

Importantly, in our BLASTP-searches, we found in six genome sequences of *C. sporogenes* isolates a TpeE-family holin that is translationally fused to its endolysin (**Figure 5B**; GenBank WP_163247266.1, strains FDAARGOS_1471, IFR 18/154, FT236, 1779, IFR 18/149, 2113/01). As all endolysins of this system are fused to a holin, this system thus cannot transport anything else than the one fused endolysin per holin, and continued transport is obviously not possible, nor is it required. This confirms the above described “single event” hypothesis, which claimed that holins do not need to transport more than one substrate and therefore can afford to be inactivated after the translocation event. This fusion also confirms that the substrate is transported when associated with the C-terminus of the holin, and agrees with the idea that the C-terminus with the bound substrate flips from cis to trans at the destabilized membrane without any larger hole formation. In case of the holin-endolysin fusion, the bound cargo endolysin may not need to be released from the holin after transport to fulfill its lytic function, especially if the C-terminus of the holin domain reaches into the cell wall, and the holin thus may serve as membrane anchor. However, it could also be that the holin is cleaved off or degraded after transport, resulting in a release of the endolysin.

In case of holins with multiple TMHs, the transport mechanism may be similar to that of BhlA family holins. In fact, many of these holins are predicted to possess C-terminal APHs, and these holins also have often unusual TMHs. Future research will need to address all these aspects, which hopefully will clarify the mechanism by which holins can transport folded endolysins or even very large bacteriocins and toxins without causing cellular lysis.

## Abbreviations:

SAR: – signal anchor release;
PspA: – pneumococcal surface protein A;
LCTs: – large clostridial toxins;
PaLoc: – pathogenicity locus;
CROP: – combined repetitive oligopeptide;
SH3b: – bacterial SH3;
Bhl: – bacteriocin-related holin-like;
Tat: – twin-arginine translocation;
TMH: – transmembrane helix;
APH: – amphipathic helix.
